# Four weeks of romosozumab treatment increases trabecular bone volume and differentially improves cortical bone in young and aging mice with chronic kidney disease

**DOI:** 10.1007/s00223-026-01577-9

**Published:** 2026-07-21

**Authors:** Corinne E. Metzger, Allison R. Kolhouse, Joana Santamaria Vilchis, Samantha P. Tippen, Landon Y. Tak, Alec N. LaPlant, Olivia N. White, Joseph M. Wallace, Matthew R. Allen

**Affiliations:** 1 Department of Anatomy Cell Biology and Physiology, Indiana University School of Medicine, 635 Barnhill Drive, MS 5045, Indianapolis, IN 46202 USA; 2 Weldon School of Biomedical Engineering, Purdue University, Indianapolis, IN 46202 USA; 3 Roudebush Veterans Administration Medical Center, Indianapolis, IN 46202 USA; 4 Department of Medicine, Division of Nephrology, Indiana University School of Medicine, Indianapolis, IN 46202 USA

**Keywords:** Chronic kidney disease, Romosozumab, Cortical porosity, Aging

## Abstract

Chronic kidney disease (CKD) leads to significant bone loss and high risk of fractures. Fractures are even more prevalent in the aging population with CKD. Cortical bone is particularly impacted in CKD due to cortical thinning and the development of cortical porosity. The goal of this study was to assess the impact of romosozumab, a bone anabolic therapy, on cortical bone in young and aging mice with adenine-induced CKD. Male C57Bl6/J mice, aged 16- and 66-weeks, were given 0.2% dietary adenine to induce CKD. Eight weeks after induction of CKD, a subset of young and aging adenine-CKD mice were given romosozumab (10 mg/kg, 1x/week for 4 weeks); the study also included age-matched healthy controls. At the study endpoint, all adenine-CKD mice had high blood urea nitrogen and parathyroid hormone compared to controls. Romosozumab-treated groups of both ages had higher trabecular bone volume due to increased trabecular thickness, indicating an anabolic effect of therapy. Romosozumab-treated young mice had higher midshaft femur cortical bone area and cortical thickness, but there were no differences in cortical porosity compared to untreated adenine. Aging mice had no differences in cortical thickness, but cortical pore number was lower with romosozumab treatment. Four-point-bending mechanical tests showed 20% higher ultimate force with romosozumab treatment in both ages, statistically different from untreated adenine in the young cohort. These data demonstrate positive cortical bone effects from four weeks of romosozumab treatment in both young and aging mice with established CKD.

## Introduction

Chronic kidney disease (CKD) impacts 1 in 7 adults within the United States [1] and 10% of the global population [2]. A large proportion of CKD patients (~ 35% of the US population) are over the age of 65 [1]. CKD leads to high rates of fracture [3–5] and increased post-fracture complications including higher post-fracture mortality [6–8]. Importantly, fracture rates are higher at every stage of CKD in patients over age 65 compared to patients with matched kidney function aged 40–65 [9]. One large cohort study in hemodialysis patients found patient age to be the greatest predictor of new fracture, both hip fracture and any fracture, in men and women [10]. Treating skeletal fragility in CKD remains a key concern for the health and quality of life in CKD patients; furthermore, assessing whether treatments are effective across the age spectrum impacted by CKD is needed.

CKD primarily targets cortical bone through cortical thinning and the development of cortical porosity. Clinical studies show that cortical bone deterioration occurs rapidly in CKD patients [11]. These effects can be modeled in preclinical studies as animal models of CKD also show significant cortical bone deterioration, most notably through the development of cortical porosity [12–14]. Since CKD is often silent and not diagnosed until later stages, treatment initiation often begins after bone is already lost. Therefore, treatments that build cortical bone, rather than prevent loss, are particularly intriguing.

Currently, anabolic therapies for bone modulate parathyroid hormone (teriparatide) or parathyroid hormone-related peptide (abaloparatide) or work via inhibiting sclerostin. PTH/PTHrP treatments increase overall bone turnover and, due to the high prevalence of secondary hyperparathyroidism in CKD, its therapeutic use in CKD is uncertain. Romosozumab, a monoclonal antibody against sclerostin, is efficacious as a bone anabolic therapy for osteoporosis [15–17] and has the additional benefit of mildly suppressing bone resorption [18, 19]. Analyses from clinical trials of romosozumab for osteoporosis have found romosozumab to be safe and effective in postmenopausal women with mild-to-moderate CKD [20, 21]. Safety in more advanced CKD stages, including hemodialysis or transplant patients, has been tested in smaller studies with positive outcomes with limited exploration into bone outcomes [22–24]. Whether romosozumab treatment can improve cortical bone parameters in CKD, particularly in aging, has not been fully explored. In the current study, our goal was to assess the impact of romosozumab treatment on cortical bone parameters and bone mechanical properties in young and aging mice with CKD. We hypothesized that 4 weeks of romosozumab treatment would result in higher cortical thickness, lower cortical porosity, and improved mechanical properties in CKD mice of both ages.

## Methods

### Animals

**Fig. 1:**
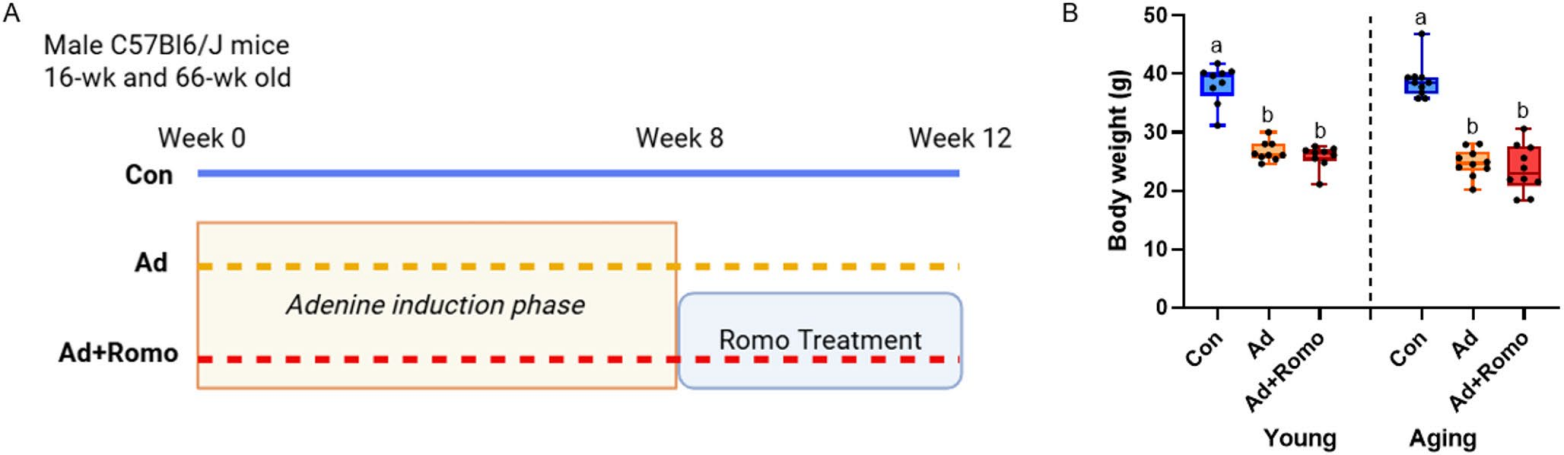
Study timeline and study endpoint body weight. **A** Schematic of the study design. Adenine induction phase = 0.2% adenine in diet. Romo treatment = dose of 10 mg/kg, 1x/week. **B** Body weight was lower in all adenine-CKD groups compared to age-matched controls. Bars not sharing the same letter are statistically different (*p* < 0.05) from the Tukey post-hoc following a one-way ANOVA within the age-matched group. Data are represented as a box and whisker plot (minimum value, lower quartile, median, upper quartile, maximum value) with all individual data points plotted

Male C57Bl/6J mice were obtained from Jackson Laboratories (JAX stock #000664, Bar Harbor, ME, USA) at 15 weeks of age or 65 weeks of age and group housed in age-matched groups (3–5/cage) at an institutionally approved animal facility with 12 h light/dark cycles. After one week of acclimatization to the facility, all mice were switched to either the control or adenine diet. The control diet (Teklad Diets [TD.150303]; Inotiv, Madison, WI, USA) was casein-based with 0.6% calcium and 0.9% phosphorus. Control mice (Con) remained on this diet for the entire 12-week study duration. Adenine-induced CKD mice (Ad) received the same casein-based diet with 0.2% adenine (AD; Teklad Diets [TD.170948]; Inotiv) for a 6-week induction period followed by the control diet for the remaining 6 weeks of the protocol. In week 8, romosozumab-treated mice (Ad + Romo) received weekly subcutaneous injections of romosozumab (10 mg/kg; Amgen, Thousand Oaks, CA) for 4 weeks (Fig. 1A). Body weight was measured weekly, and animals were monitored for health daily. After 12 weeks, mice were fully anesthetized via vaporized inhaled isoflurane and humanely euthanized via exsanguination followed by thoracotomy. Body weight, serum blood urea nitrogen, and serum parathyroid hormone in the young Con and Ad groups are previously published in a different study with a different hypothesis [25], but all bones were scanned and analyzed independently for this study. As previously published, aging adenine mice have higher attrition rate than young adenine mice [26]. During the 4-week treatment phase (weeks 8–12), n = 2 animals in the Ad and Ad + Romo groups of the aging cohort were found dead or euthanized due to rapid weight loss. No animals were lost in the young cohort. Final sample sizes were n = 9/group. All animal procedures were approved by the Indiana University School of Medicine Institutional Animal Care and Use Committee prior to the initiation of experimental protocols, and methods were carried out in accordance with relevant guidelines and regulations.

### Serum assays

Cardiac blood collected at the time of termination was used to measure serum blood urea nitrogen (BUN) (BioAssay Systems, Hayward, CA, USA), serum phosphorus (Pointe Scientific, Canton, MI, USA), and serum calcium (Pointe Scientific) via colorimetric assays. Serum parathyroid hormone (Immutopics Quidel, San Diego, CA, USA), serum P1NP, and TRAcP 5b (Euroimmun US, Mountain Lakes, NJ, USA) were measured via ELISA. All serum assays were performed in duplicate following manufacturer protocols.

### Micro-computed tomography

The left distal femora and left tibiae were scanned 3/scan in a multi-bone holder as previously described [27] on a SkyScan 1272 CMOS system (Bruker, Billerica, MA, USA) with a 0.5 aluminum filter and a 7 µm voxel size. Scans were reconstructed and rotated to a standard orientation utilizing SkyScan software (NRecon, Dataviewer). Trabecular bone of the distal femur was analyzed in a 1 mm region starting proximal to the growth plate in the distal femur with a binary threshold of 115–255. Cortical bone properties were analyzed in a 1 mm region in the femoral midshaft. Volumetric cortical porosity and pore network data (pore number, average pore length, average distance between pores) were assessed from hand drawn regions of interest (tracing the periosteal and endosteal surfaces) and measured with an inverse binary threshold of 115–0. Cortical bone area was measured from the same region of interest but analyzed with a binary threshold of 115–255. Finally, cortical thickness was measured with a flooded threshold (0–255) to obtain an average width from the endocortical to periosteal edges without accounting for porosity within the region as previously described [25]. The left midshaft tibia was analyzed for total area, cortical bone area, and cortical thickness in 5 contiguous slices. Total area and cortical bone area were analyzed with a binary threshold of 115 to 255 and cortical thickness was measured with a flooded threshold (0 to 255).

### Four-point bending of the tibia

Right tibiae were wrapped in PBS-soaked gauze and frozen. Samples were thawed and scanned in the same method as described above. Four-point bending was performed (TA Instruments, New Castle, DE, USA). The medial surface of the bone was placed in tension on two metal supports located ± 9 mm from the mid-diaphysis testing site, and the upper supports were centered on the bone with a span of ± 3 mm. Specimens were loaded to failure at a rate of 0.025 mm/sec, producing a force–displacement curve for each sample. Structural mechanical properties (ultimate force, stiffness, total displacement) were obtained directly from the force–displacement curves using a MATLAB code, while estimated material properties (modulus, toughness) were derived from force–displacement curves and geometric properties noted above using standard beam-bending equations for four-point bending.

### Statistical analyses

Data were analyzed within the age-matched cohort. All data were tested to determine if data met standards for homogeneity of variances (Levene’s test). If data met homogeneity standards, a one-way ANOVA was completed. If statistically significant (*p* < 0.05), a Tukey post-hoc test was completed. When data did not meet standards for homogeneity of variances (Levene’s test *p* < 0.05), a Kruskal–Wallis test (described as KW test in results) was completed followed by a Dunn’s post hoc with Bonferroni correction when applicable. All statistics were completed with IBM SPSS Statistics version 31 (IBM, Armonk, NY, USA).

## Results

### Markers of CKD were largely unaffected by 28 days of romosozumab treatment

**Fig. 2:**
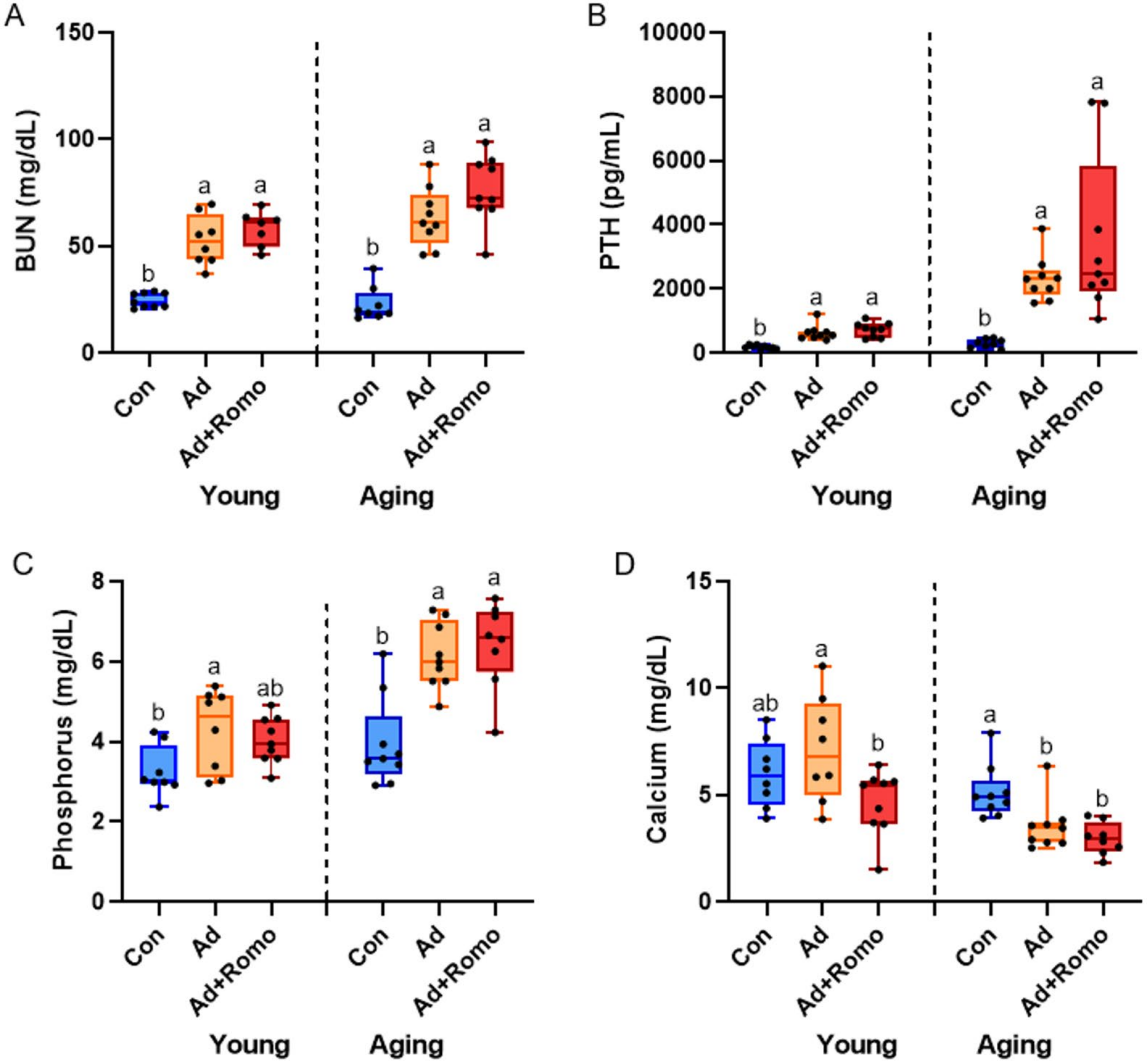
Serum markers of chronic kidney disease. **A** Serum BUN was higher in adenine groups regardless of treatment in young and aging mice. **B** Serum PTH was higher in adenine groups in both young and aging cohorts. **C** Serum phosphorus was higher in young Ad compared to Con with Ad + Romo not different from either group. In the aging cohort, serum phosphorus was higher in both adenine groups compared to Con. **D** Serum calcium was lower in Ad + Romo compared to Ad in the young cohort. In the aging cohort, serum calcium was lower in both adenine groups compared to Con. Bars not sharing the same letter are statistically different (*p* < 0.05) from the Tukey post-hoc following a one-way ANOVA within the age-matched group. Data are represented as a box and whisker plot (minimum value, lower quartile, median, upper quartile, maximum value) with all individual data points plotted

Body weight was lower in adenine mice compared to age-matched controls in both age groups with no differences due to Romo treatment (*p* < 0.001 for both ages; Fig. 1B). Serum BUN (KW test for young) and PTH (KW test for aging) were higher in adenine mice in both age groups with no statistical differences in Ad + Romo vs. untreated Ad in either age (*p* < 0.001 for all; Fig. 2A and B). In the young cohort, serum phosphorus was higher in untreated Ad vs. Con with Ad + Romo not different from either group (*p* = 0.030). In the aging cohort, serum phosphorus was higher in both adenine groups compared to age-matched control (*p* < 0.001; Fig. 2C). Serum calcium was lower in Ad + Romo vs. Ad in the young cohort (*p* = 0.044), but both adenine groups were lower than age-matched control in the aging cohort (*p* < 0.001; Fig. 2D). Overall, adenine-CKD mice demonstrated reduced kidney function (high BUN) and hyperparathyroidism with no impact of treatment.

### Aging CKD mice treated with romosozumab had higher P1NP than untreated CKD

**Fig. 3:**
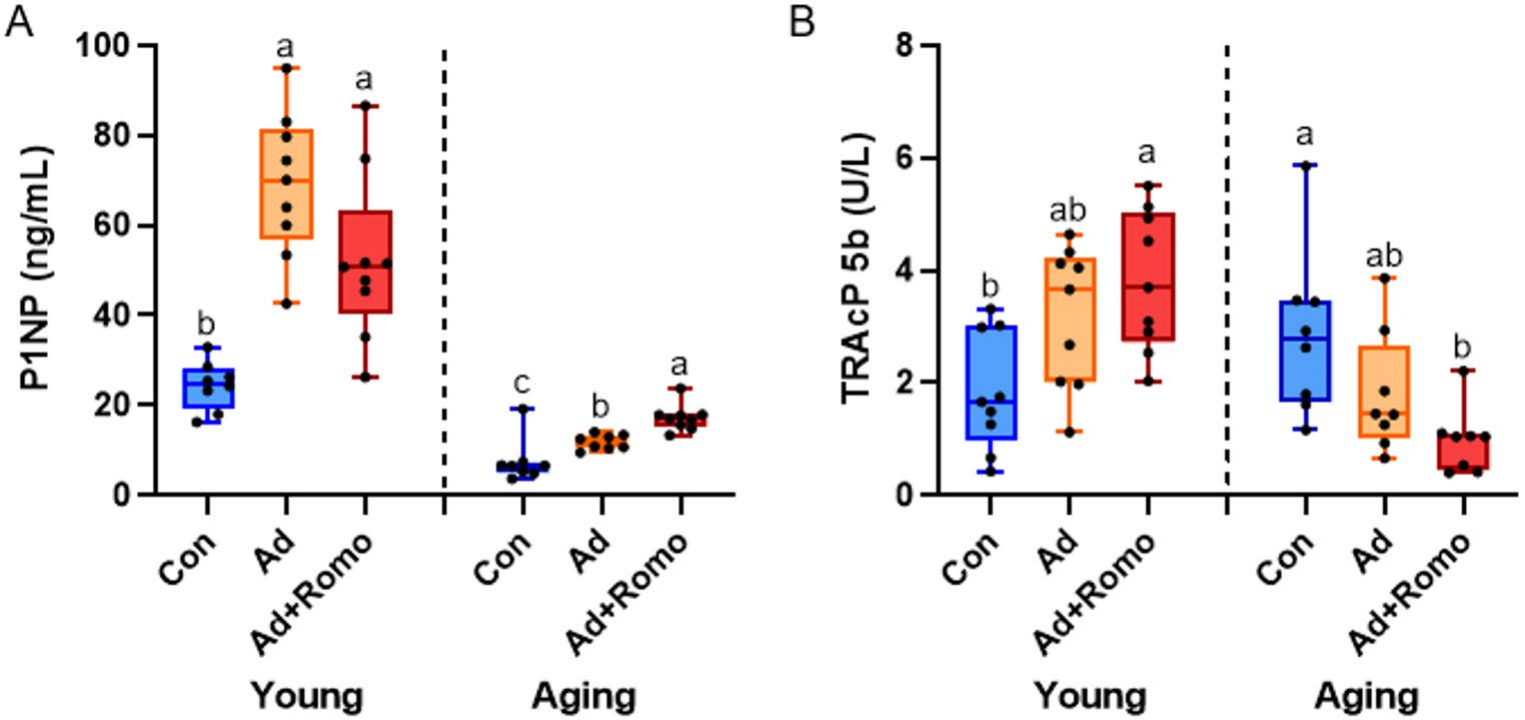
Serum markers of bone turnover. **A** Serum P1NP was higher in CKD groups compared to Con in young. In aging, P1NP was higher in Ad + Romo compared to Ad. **B** Serum TRAcP 5b was higher in Ad + Romo compared to Con in young. In aging, serum TRAcP 5b was lower in Ad + Romo vs. Con. Bars not sharing the same letter are statistically different (*p* < 0.05) from the Tukey post-hoc following a one-way ANOVA within the age-matched group. Data are represented as a box and whisker plot (minimum value, lower quartile, median, upper quartile, maximum value) with all individual data points plotted

P1NP, a circulating marker of bone formation, was different in both young and aging groups (*p* < 0.001 for both), with higher P1NP in Ad in both ages. Aging Ad + Romo had higher P1NP than untreated Ad (Fig. 3A). Serum TRAcP 5b, a serum marker of osteoclasts, was also different in both ages (*p* = 0.006 in young, *p* = 0.01 in aging). In the young cohort, Ad + Romo had higher TRAcP 5b than Con. In the aging cohort, Ad + Romo was lower than Con. In both cohorts, TRAcP 5b was not different between Ad and Ad + Romo groups (Fig. 3B).

### Romosozumab treatment resulted in higher trabecular bone volume at the distal femur at both ages primarily due to greater trabecular thickness

**Fig. 4:**
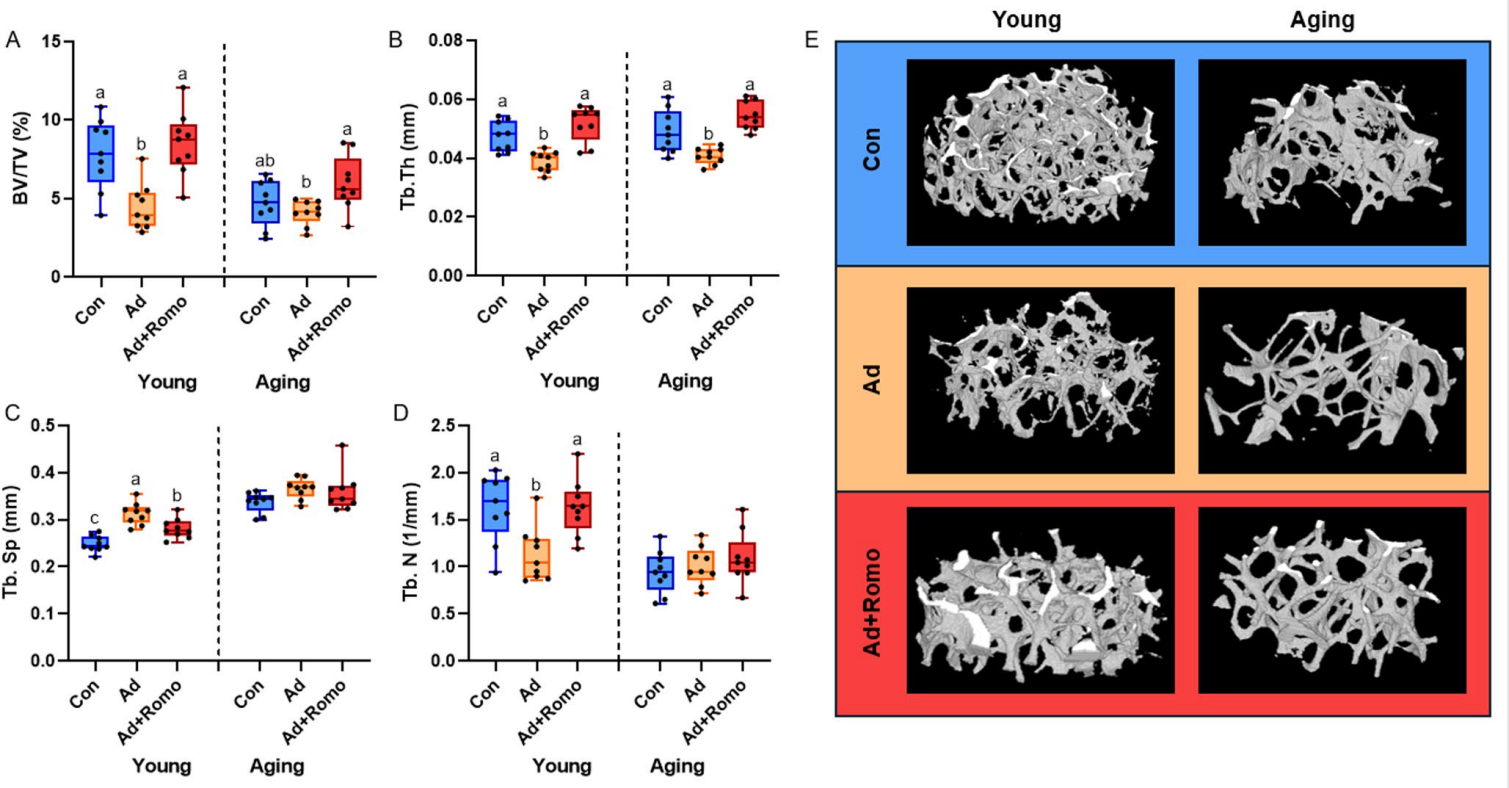
Trabecular bone microarchitecture from the distal femur metaphysis. **A** Trabecular bone volume was lower in Ad vs. Ad + Romo and Con in the young cohort. In the aging cohort, Ad + Romo was higher than Ad. **B** Trabecular thickness was lower in untreated adenine compared to all other groups in both the young and the aging cohort. **C** Trabecular separation was highest in Ad followed by Ad + Romo and then Con in the young cohort. There were no statistical differences in the aging cohort. **D** Trabecular number was lower in Ad vs. both Con and Ad + Romo in the young cohort. There were no statistical differences in trabecular number in the aging cohort. **E** Representative images of trabecular bone volume. Images represent the sample closest to the group mean for each group for young (left column) and aging (right column). Bars not sharing the same letter are statistically different (*p* < 0.05) from the Tukey post-hoc following a one-way ANOVA within the age-matched group. Data are represented as a box and whisker plot (minimum value, lower quartile, median, upper quartile, maximum value) with all individual data points plotted

In the young cohort, trabecular bone volume was lower in untreated Ad vs. both Con and Ad + Romo (*p* < 0.001; Fig. 4A). Trabecular thickness followed the same pattern with untreated Ad lower than both Con and Ad + Romo. Ad + Romo was not statistically different from Con (*p* < 0.001; Fig. 4B). Trabecular separation was highest in Ad followed by Ad + Romo and then Con (*p* < 0.001; Fig. 4C). Trabecular number was lower in untreated Ad vs. both Con and Ad + Romo (*p* = 0.003; Fig. 4D).

In the aging cohort, trabecular bone volume was higher in Ad + Romo compared to Ad with Con not different from either group (*p* = 0.023; Fig. 4A). Trabecular thickness (KW test) was lower in untreated Ad compared to both Con and Ad + Romo (*p* < 0.001; Fig. 4B). Neither trabecular separation nor trabecular number showed statistical difference between groups in the aging cohort (*p* = 0.130, *p* = 0.437; Fig. 4C and D). Higher trabecular bone volume due to greater trabecular thickness demonstrates the anabolic effect of treatment despite CKD in both young and aging cohorts.

### Cortical bone area and cortical thickness in the femur and tibia were higher in the romosozumab treatment group in the young, but not the aging cohort

**Fig. 5:**
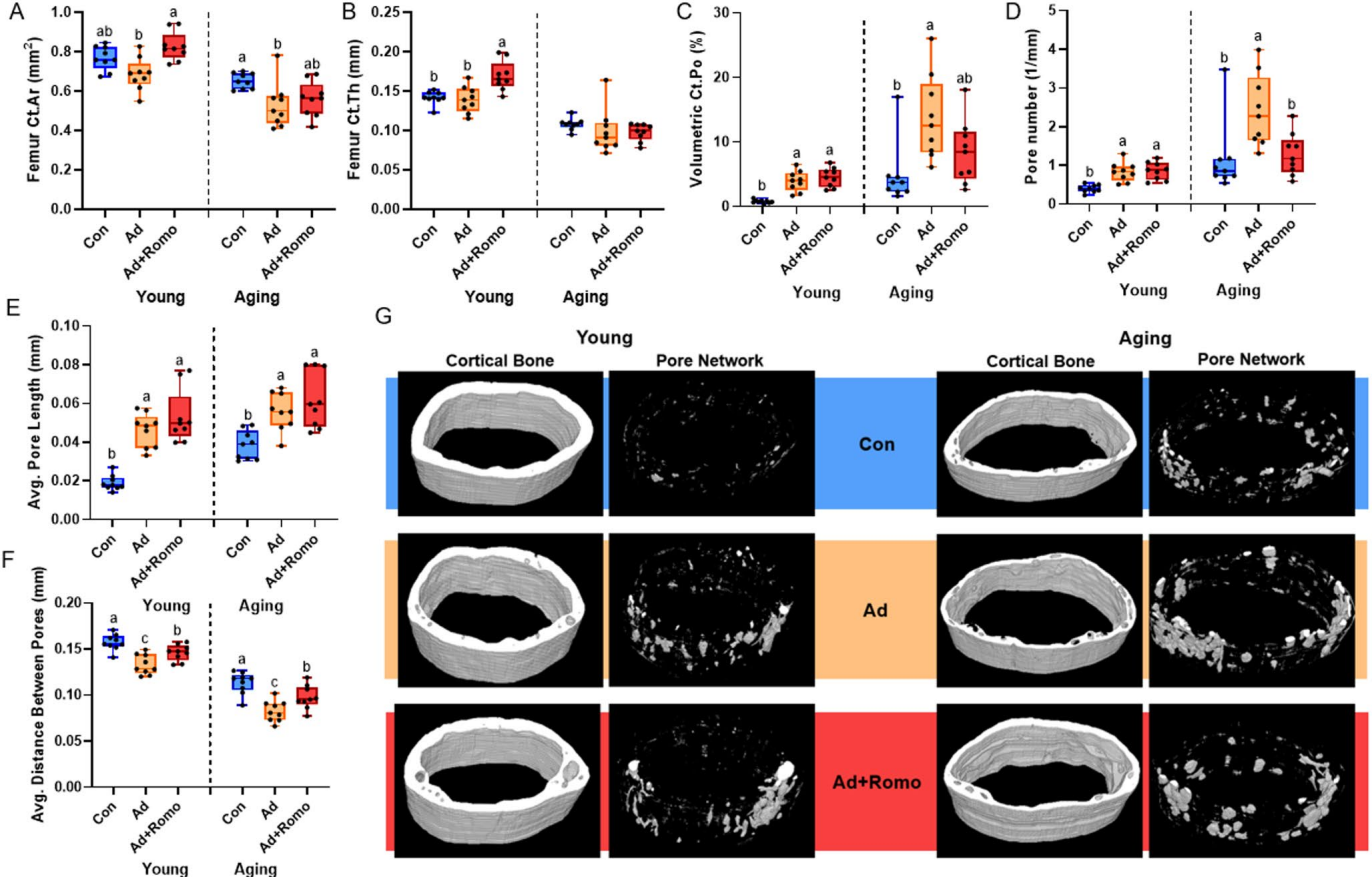
Midshaft femur cortical microarchitecture. **A** Cortical bone area was higher in Ad + Romo compared to Ad in the young cohort. In the aging cohort, Ad was lower than Con with Ad + Romo not different from either group. **B** Cortical thickness was higher in Ad + Romo compared to both Con and untreated Ad in the young cohort. There were no differences in cortical thickness in the aging cohort. **C** Volumetric cortical porosity was higher in both adenine groups compared to Con in the young cohort. In the aging cohort, volumetric cortical porosity was higher in Ad vs. Con with Ad + Romo not different from either group. **D** Pore number was higher in both adenine groups compared to Con in the young cohort. In aging, pore number was higher in untreated Ad compared to both Con and Ad + Romo. **E** In both cohorts, the average length of the pore was higher in adenine compared to Con with no effect of treatment. **F** In both cohorts, the average distance between pores was lowest in untreated adenine followed Ad + Romo with Con have the greatest distance. **G** Representative images of cortical bone volume and the pore network in the midshaft femur in all groups in young (left two columns) and aging (right two columns). Images represent the sample closest to the group mean. Bars not sharing the same letter are statistically different (*p* < 0.05) from the Tukey post-hoc following a one-way ANOVA within the age-matched group. Data are represented as a box and whisker plot (minimum value, lower quartile, median, upper quartile, maximum value) with all individual data points plotted

In the midshaft femur in both cohorts, there were no statistical differences in total bone area (*p* = 0.883 in young, *p* = 0.552 in aging). In the young cohort, cortical bone area (*p* < 0.001) was higher in Ad + Romo compared to untreated Ad with Con not different from either group (Fig. 5A). Additionally, cortical thickness in the young cohort (*p* < 0.001) was higher in Ad + Romo compared to both Con and untreated Ad (Fig. 5B). In the aging cohort, cortical bone area (*p* = 0.014) was lower in untreated Ad compared to Con with Ad + Romo not different from either group (Fig. 5A). There were no differences in cortical thickness (KW test) in the aging cohort (*p* = 0.058; Fig. 5B).

**Fig. 6:**
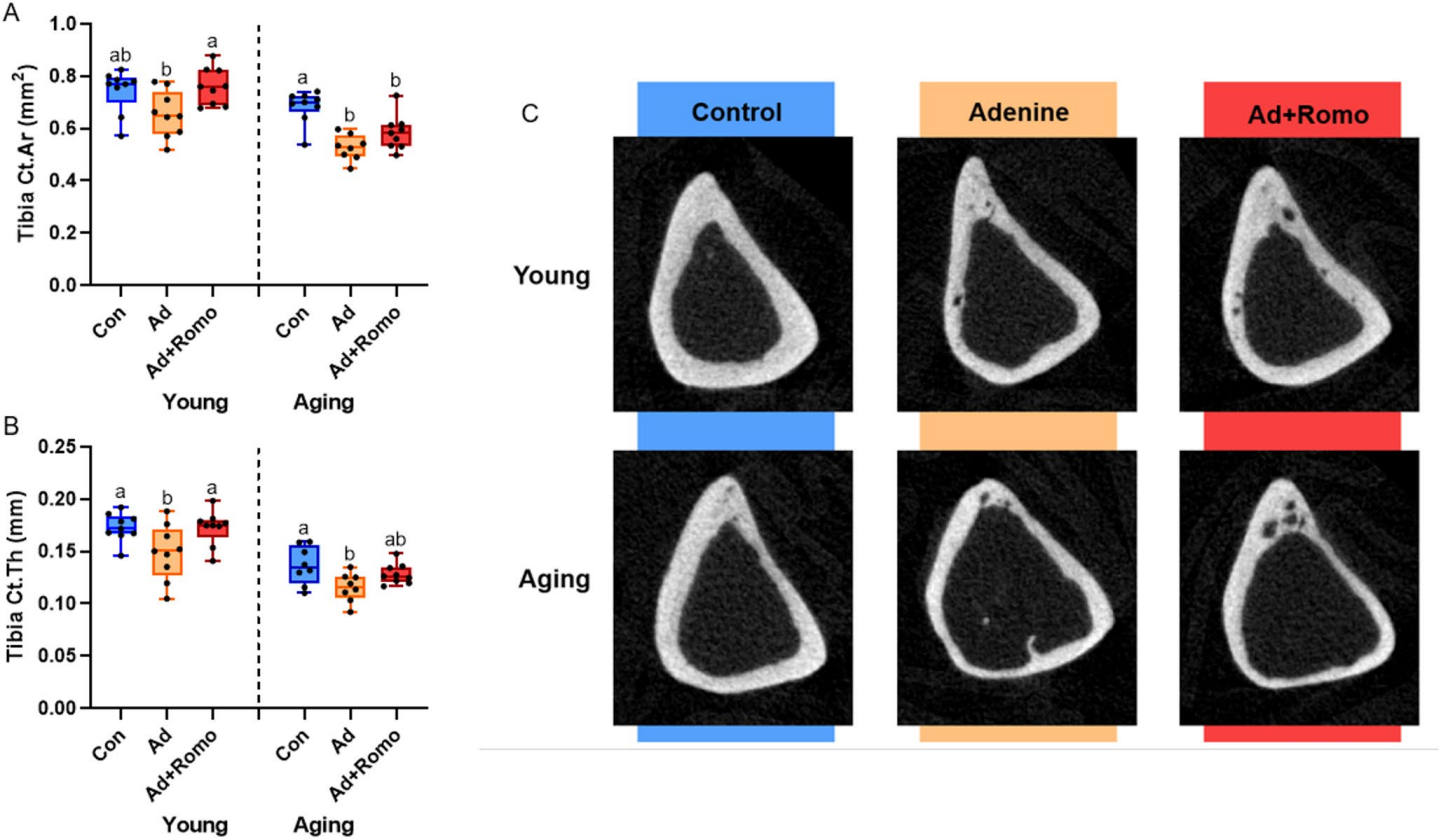
Tibia midshaft cortical microarchitecture **A** In the young cohort, tibia cortical bone area was higher in Ad + Romo compared to Ad. In the aging cohort, both adenine groups were lower than Con. **B** Cortical thickness was lower in untreated Ad compared to both Con and Ad + Romo in the young cohort. In the aging cohort, untreated adenine was lower than Con with Ad + Romo not different from either group. **C** Representative images of the midshaft tibia from micro-CT of treatment groups in young and aging. Images represent the sample closest to the group mean. Bars not sharing the same letter are statistically different (*p* < 0.05) from the Tukey post-hoc following a one-way ANOVA within the age-matched group. Data are represented as a box and whisker plot (minimum value, lower quartile, median, upper quartile, maximum value) with all individual data points plotted

At the midshaft tibia, total area was not different in either cohort (*p* = 0.651 for young, *p* = 0.142 in aging). In the young cohort, cortical bone area (*p* = 0.021) was higher in Ad + Romo vs Ad with Con not different from either group (Fig. 6A). Cortical thickness (*p* = 0.021) was lower in untreated Ad compared to both Con and Ad + Romo in the young cohort (Fig. 6B). In the aging cohort, cortical bone area (*p* < 0.001) was lower in both adenine groups compared to control with no difference due to treatment (Fig. 6A). Cortical thickness (*p* = 0.024) was lower in untreated Ad compared to Con with Ad + Romo not different from either group in the aging cohort (Fig. 6B). Figure 6C shows representative micro-CT images of the tibia midshaft. Taken together, younger mice had greater effects of romosozumab treatment on cortical bone structural parameters at both bone sites.

### Romosozumab treatment resulted in lower cortical pore number at the femur midshaft in aging, but not young adenine-CKD mice

In young mice at the midshaft femur, volumetric cortical porosity (KW test; *p* < 0.001) was higher in both adenine groups compared to Con (Fig. 5C). Likewise, average pore length and pore number (KW test; *p* < 0.0001 for both) showed the same pattern with adenine groups higher than Con and no effect of romosozumab treatment (Fig. 5D and E). The average distance between pores (*p* < 0.001) was lowest in untreated Ad followed by Ad + Romo and then Con (Fig. 5F).

In aging mice, volumetric cortical porosity (*p* = 0.007) was higher in untreated Ad compared to Con (Fig. 5C). The Ad + Romo group was not different from either group. Pore number (*p* = 0.005) was lower in Ad + Romo compared to untreated Ad, not statistically different from Con (Fig. 5D). Average pore length (*p* < 0.001) was higher in both adenine groups compared to Con (Fig. 5E). Average distance between pores (*p* < 0.001) was lowest in untreated Ad with Ad + Romo statistically higher than Ad, but lower than Con (Fig. 5F). Figure 5G shows representative images of cortical bone volume and the pore network. Overall, pore network parameters were not impacted by romosozumab treatment in young mice while pore number was lower due to treatment in aging mice.

### Romosozumab treatment had a greater effect on mechanical properties in the tibia in young vs. aging mice

**Fig. 7:**
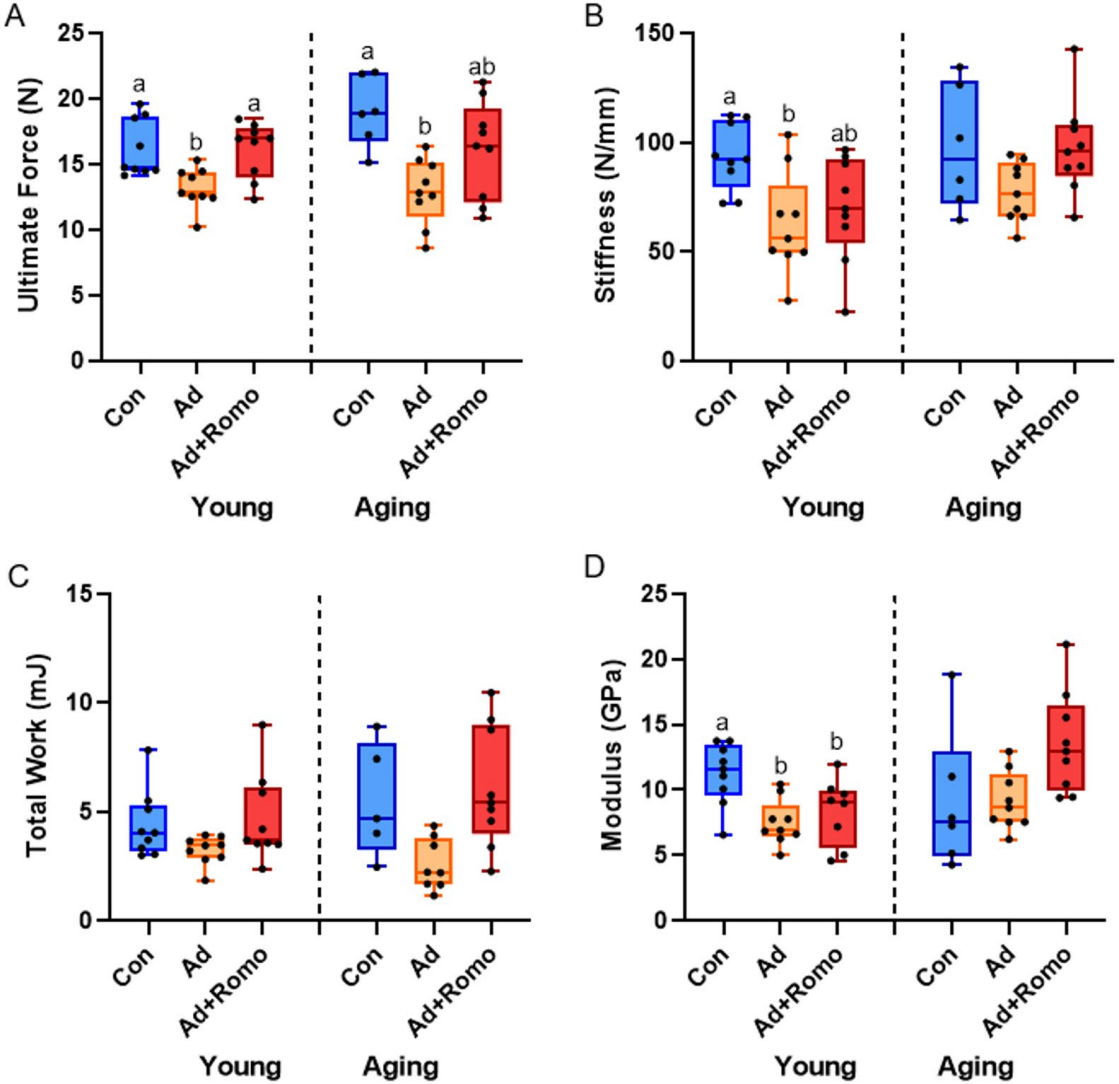
Mechanical properties from the tibia four-point-bend test. **A** Ultimate force was lower in Ad compared to Con and Ad + Romo in the young cohort. In the aging cohort, untreated Ad was lower than Con with Ad + Romo not different from either group. **B** In the young cohort, stiffness was lower in Ad vs. Con with Ad + Romo not different from either group. In the aging cohort, there were no statistical differences between groups. **C** There were not statistical differences in total work in either age cohort. **D** Modulus was lower in both adenine groups compared to Con in the young cohort. There were no differences in the aging cohort. Bars not sharing the same letter are statistically different (*p* < 0.05) from the Tukey post-hoc following a one-way ANOVA within the age-matched group. Data are represented as a box and whisker plot (minimum value, lower quartile, median, upper quartile, maximum value) with all individual data points plotted

In young mice, ultimate force (*p* = 0.005) was lower in untreated Ad compared to both Con and Ad + Romo. Ad + Romo was not different from Con (Fig. 7A). Stiffness (*p* = 0.013) was higher in Con compared to untreated Ad with Ad + Romo not different from either group (Fig. 7B). Modulus (*p* = 0.005) was also different with Con higher than both adenine groups (Fig. 7D). There were no statistical differences in total displacement (*p* = 0.492), total work (*p* = 0.086; Fig. 7C), or toughness (*p* = 0.506).

In aging mice, ultimate force (*p* = 0.004) was different between groups with untreated Ad lower than Con. Ad + Romo was not different from either group (Fig. 7A). Modulus showed a statistical difference in the one-way ANOVA (*p* = 0.037), but no post hoc differences were detected (Fig. 7D). In the aging cohort, there were no statistical differences in stiffness (p = 0.099), total displacement (p = 0.543), total work (*p* = 0.086), or toughness (*p* = 0.170). When compared together, ultimate force was ~ 21–24% higher in romosozumab-treated CKD mice compared to age-matched untreated mice in both cohorts, but this only reached statistical difference in the young cohort.

## Discussion

This study documents that four weeks of romosozumab treatment in mice with established CKD improved cortical bone parameters with more pronounced benefits in young mice. Interestingly, romosozumab treatment increased cortical thickness in young mice, but not aging mice, while the treatment resulted in lower pore number only in the aging cohort. Romosozumab treatment had a robust effect on trabecular bone volume due to higher trabecular thickness in both ages.

Prevention of skeletal fragility in CKD is a key clinical goal. However, finding effective and safe treatments in the setting of reduced renal function is challenging. Bisphosphonates, key mainstays of osteoporosis treatment for decades, have been viewed as potentially concerning for renal toxicity with more recent evidence showing a modest increased risk of CKD progression with bisphosphonate use in moderate-to-severe CKD [28]. Rodent models of CKD have shown positive effects on remodeling suppression yet worsened cortical bone parameters with different dosing schemes with bisphosphonates [29]. Cortical bone loss in CKD is linked with secondary hyperparathyroidism [11]; therefore, control of PTH remains essential in CKD. While non-pharmacological suppression of PTH with calcium water supplementation in rodent models successfully mitigates or reverses cortical bone deterioration [30], pharmacological PTH treatment has more modest effects [31]. Clinical trials with calcimimetics aimed at lowering PTH have minimal efficacy in fracture reduction [32]. Together, these data show the complexity of treating bone in CKD and highlight the need for more treatment options. Romosozumab is an attractive option due to its dual actions in increasing bone formation and mildly suppressing bone resorption as well as its prominent positive effects on cortical bone.

Romosozumab, a monoclonal antibody against sclerostin, is approved for treatment of severe osteoporosis. Sub-analyses of clinical trials in post-menopausal women with osteoporosis have assessed those with mild-to-moderate CKD and shown increased bone mineral density and reduction in new vertebral fractures [20, 21]. No full clinical trials have been completed with CKD patients in more advanced stages of CKD or those on maintenance hemodialysis. Several smaller retrospective or observational studies in hemodialysis or kidney transplant patients have shown increased bone mineral density with romosozumab treatment [23, 24, 33]. Studies with pre-clinical models have shown romosozumab treatment improved cortical bone area in a combined model of adenine-CKD with hyperglycemia via streptozotocin [34, 35]. With a different anti-sclerostin antibody, rats with progressive CKD had positive skeletal effects of the anti-sclerostin treatment only when combined with PTH suppression [36]. Our primary goal of the current work was to study the impact of romosozumab in CKD-induced bone loss in an aging preclinical model where cortical bone deficits are more pronounced than in young, skeletally mature rodents [26].

In our current study, adenine-CKD mice of both ages had lower trabecular bone volume due to adenine-induced CKD, but four weeks of romosozumab treatment resulted in higher trabecular bone volume—62% greater in young and 37% greater in aging compared to age-matched untreated adenine-CKD. In both groups, romosozumab-treated adenine mice had trabecular bone volume that was not statistically different from control levels and even marginally higher (24% higher) than controls in the aging cohort. These changes were primarily attributed to higher trabecular thickness due to romosozumab treatment regardless of age. Therefore, despite aging-related negative effects, romosozumab still exerted a clear anabolic effect on trabecular bone parameters. This was supported by serum P1NP, a marker of bone formation, which was higher in the aging romosozumab-treated mice compared to untreated adenine. Interestingly, this effect was not seen in the young mice. Additionally, serum TRAcP 5b, a serum marker of osteoclasts, trended in different directions in young vs. aging. Since both serum markers only show a single time point, it is difficult to interpret these results; however, the microCT changes of bone reflect the full time course of treatment and demonstrate a clear anabolic response.

In cortical bone at the midshaft femur, there was a more pronounced effect of adenine-CKD in aging mice compared to young. Aging mice with CKD had 20% lower cortical bone area compared to age-matched controls while young CKD mice had 10% lower cortical bone area compared to age-matched controls. Romosozumab treatment had a greater effect in young adenine-CKD mice compared to aging with 18% higher cortical bone area in treated vs. untreated adenine in young, but a ~ 6% difference between those groups in the aging cohort. Cortical thickness was higher due to romosozumab treatment in the young cohort only, ~ 18% higher than both control and untreated adenine. In the midshaft tibia, the impact of romosozumab treatment was similar with higher cortical bone area and cortical thickness in young adenine-CKD mice, but not in aging adenine-CKD mice. Total bone area was not impacted by treatment in either group, indicating the romosozumab-induced changes were not primarily due to periosteal expansion. These data demonstrated a more pronounced effect of romosozumab treatment in the cortical bone of younger mice compared to aging mice. This is consistent with age-related blunted anabolic responses to PTH [37] and mechanical loading [38–40]. Importantly, in young mice, these romosozumab-induced improvements occurred despite the high circulating PTH which is a primary signal for cortical bone deterioration. Interestingly, the aging cohort had numerically higher values of circulating PTH in adenine-CKD mice, which could potentially relate to the less robust effect of treatment on cortical thickness in this cohort.

One interesting finding of our study was a differential response in cortical porosity in young vs. aging. Consistent with our previous work, the aging adenine-CKD mice had numerically higher cortical porosity (~ 14%) compared to young adenine mice (~ 4%). Romosozumab treatment had no effect on overall cortical porosity or pore number in young mice; however, in aging mice, romosozumab treatment resulted in a 61% lower pore number compared to the untreated group (and a non-statistically different 48% lower volumetric porosity). Due to our timing of treatment after mice already had established disease, we cannot say whether the treatment reduced/reversed porosity or if it prevented the development of further porosity, but this same effect was not seen in the young cohort indicating a unique impact of romosozumab on intracortical bone in the aging cohort. Previous work from our lab has only shown a reversal and/or infilling of existing pores with non-pharmacological suppression of parathyroid hormone via calcium water supplementation [30]. With the same treatment timing, treatment with a calcimimetic, a clinical analog of PTH suppression therapy, did not reverse or infill porosity, but did mitigate the expansion of existing pores and development of pores [31]. Mechanisms to reduce cortical porosity are attractive due to the high likelihood of bone loss already being present by the time clinical treatment is initiated and the impact of cortical porosity on bone strength. Since PTH suppression therapy has also shown the ability to improve cortical porosity, pairing a calcimimetic with romosozumab treatment may be a beneficial dual therapy to improve bone via modulating cortical porosity in CKD of aging populations.

These data also demonstrate that an anabolic stimulus to bone is not sufficient to infill existing pores. In the young mice, there were no differences in cortical porosity in the adenine mice regardless of treatment despite robust improvements in trabecular bone volume, cortical bone area, and cortical thickness, which demonstrate the clear anabolic effect of the therapy. It is possible that romosozumab treatment had a greater impact in the aging cohort on porosity due to a potential age-related decline in osteoblastic activity that was stimulated by romosozumab, but we could not assess cellular differences with our experimental design. Previous work in our lab has shown that anti-resorptive therapies alone, both bisphosphonates and anti-RANKL therapy, are also insufficient to reduce cortical porosity independently [29, 41]. These outcomes highlight that porosity reduction is complex and likely may involve a combination of PTH suppression, osteoclast inhibition and osteoblast stimulus (especially in aging individuals).

Both CKD young (− 20%) and aged (− 38%) cohorts had lower ultimate force compared to the control cohorts. Young romosozumab-treated adenine mice had ~ 20% greater ultimate force in the tibia compared to untreated adenine, resulting in values no difference from the healthy control. Romosozumab treatment also resulted in ~ 20% higher ultimate force in aging adenine-CKD mice, but this difference was not statistically different from untreated adenine. The greater deficit in mechanical properties in the aging adenine-CKD mice may indicate the need for combination treatment, like pairing romosozumab with a calcimimetic, to lead to more significant improvements.

One commonly cited concern of romosozumab therapy in more advanced stages of CKD is the risk of hypocalcemia. A single dose of romosozumab in grade 4 and 5 CKD resulted in hypocalcemia in some patients, but it was reported as asymptomatic in all instances [42]. After 12 months of therapy in kidney transplant patients and hemodialysis patients, cases of hypocalcemia were also asymptomatic [24, 33]. In our study, serum calcium at study endpoint was lower in the romosozumab-treated young adenine mice compared to untreated adenine, but not statistically different from healthy control levels. In aging mice, serum calcium at study endpoint was lower in both adenine-CKD groups compared to age-matched controls with no effect of treatment.

Limitations of our current study include assessing only male mice. Previous work from our lab has shown similar skeletal responses to adenine-induced CKD in male and female mice [13, 41], but the impact of age and romosozumab treatment has not been explored. Additionally, we were unable to complete histological assessments of bone turnover in this study due to insufficient samples across all cohorts. Finally, detailed assessment of kidney function was also not feasible in this study.

In conclusion, we document that four weeks of romosozumab treatment in skeletally mature and aging adenine-induced CKD mice led to robust improvement in trabecular bone volume across both ages. Cortical bone showed a differential response to romosozumab treatment with higher cortical thickness in young mice, but lower pore number in aging mice. Overall, these romosozumab-induced cortical bone structural differences were associated with positive effects on bone mechanical properties in young and aging mice with CKD.
